# A Possible Novel Anti-Inflammatory Mechanism for the Pharmacological Prolyl Hydroxylase Inhibitor 3,4-Dihydroxybenzoate: Implications for Use as a Therapeutic for Parkinson's Disease

**DOI:** 10.1155/2012/364684

**Published:** 2012-05-14

**Authors:** Shankar J. Chinta, Subramanian Rajagopalan, Abirami Ganesan, Julie K. Andersen

**Affiliations:** Buck Institute for Research on Aging, 8001 Redwood Boulevard, Novato, CA 94945, USA

## Abstract

Parkinson's disease (PD) is an age-related neurodegenerative disorder characterized in part by the preferential loss of nigrostriatal dopaminergic neurons. Although the precise etiology of PD is unknown, accumulating evidence suggests that PD involves microglial activation that exerts neurotoxic effects through production of proinflammatory cytokines and increased oxidative and nitrosative stress. Thus, controlling microglial activation has been suggested as a therapeutic target for combating PD. Previously we demonstrated that pharmacological inhibition of a class of enzymes known as prolyl hydroxylases via 3,4-dihydroxybenzoate administration protected against MPTP-induced neurotoxicity, however the exact mechanisms involved were not elucidated. Here we show that this may be due to DHB's ability to inhibit microglial activation. DHB significantly attenuated LPS-mediated induction of nitric oxide synthase and pro-inflammatory cytokines in murine BV2 microglial cells *in vitro* in conjunction with reduced ROS production and activation of NF*κ*B and MAPK pathways possibly due to up-regulation of HO-1 levels. HO-1 inhibition partially abrogates LPS-mediated NF*κ*B activity and subsequent NO induction. *In vivo*, DHB pre-treatment suppresses microglial activation elicited by MPTP treatment. Our results suggest that DHB's neuroprotective properties could be due to its ability to dampen induction of microglial activation via induction of HO-1.

## 1. Introduction

Parkinson's disease (PD) is a slowly progressive age-related neurodegenerative disorder characterized by irreversible degeneration of the dopaminergic nigrostriatal pathway, resulting in marked impairments of motor control. Although PD has been heavily researched in the last two decades, the precise etiology of the disease is still unknown. However, research in recent years has provided substantial evidence supporting the hypothesis that oxidative stress and inflammation both likely play a major role in disease pathogenesis [1–4].

A growing body of both experimental and clinical studies suggests that inflammation may contribute to neurodegeneration associated with many neurological diseases including PD [5, 6]. The first evidence for a role for inflammation in PD came from a postmortem study by McGeer and colleagues who found activated microglia and T-lymphocytes in the SNpc of a PD patient [7]. Since then, there have been numerous reports supporting a role for neuroinflammatory processes in PD pathogenesis [8–10]. In addition to the presence of activated microglia, increased levels of proinflammatory cytokines including IL1*β* and IL-6 and enzymes associated with inflammation such as inducible nitric oxide synthase (iNOS) and cyclooxygenase 2 (COX2) have been observed in the Parkinsonian brain [11, 12].

Microglia are the resident immune-competent cells in the brain that act to amplify the effects of inflammation thereby mediating ongoing cellular degeneration [13, 14]. In the event of brain damage or infection, microglia cells become activated and secrete a variety of proinflammatory mediators and other potentially neurotoxic factors which can have deleterious effects on neighboring neurons. Suppression of microglia activation has been suggested as a possible therapeutic intervention that may alleviate the progression of various neurodegenerative diseases including PD [15].

Prolyl 4-hydroxylases (PHDs) are a family of enzymes that act to hydroxylate a variety of substrates, the most well studied of which is hypoxia-inducible factor (HIF). HIF is a transcription factor that plays a major role in the regulation of cellular and systemic oxygen homeostasis [16]. It is a heterodimer consisting of a constitutively expressed *β* subunit and one of two *α* subunits, HIF-1*α* or HIF-2*α*, which are mainly regulated by oxygen. During normoxia, HIF*α* is continuously synthesized and hydroxylated on a specific proline residue by specific PHD isoforms in a cell- and tissue-specific manner [17]. Hydroxylated HIF*α* is rapidly ubiquitylated and subsequently degraded by the proteasome. Under hypoxic conditions, PHDs are prevented from hydroxylating proline residues of the HIF-1*α* protein resulting in upregulation of HIF*α* isoforms. This results in accumulation of HIF*α* in the cytosol followed by its translocation to the nucleus where it binds HIF-1*β*. The heterodimer then binds to hypoxia response elements (HREs) found on a variety of genes including heme oxygenase-1 (HO-1), transferrin receptor (TfR), and tyrosine hydroxylase (TH), resulting in their transcriptional induction [18].

Previous studies have shown that broad pharmacological inhibition of the PHDs alleviates neurodegeneration associated with stroke and hypoxic-ischemic injuries [19, 20]. Recently, studies from our own laboratory found that PHD inhibition via the broad-acting inhibitor 3,4-dihydroxybenzoate (DHB) protects against MPTP-induced neurotoxicity [21]. The exact mechanisms involved, however, were not elucidated in this previous study. Here, we report that the neuroprotective effects of DHB could be due at least in part to antiinflammatory properties of the drug. Our results demonstrate that DHB prevents microglial activation that coincides with reduced neuronal cell loss in both *in vitro* and *in vivo* models of PD. These effects may be attributable to DHB's known ability to induce increases in HO-1 levels, in turn eliciting both anti-oxidant and anti-inflammatory effects.

## 2. Materials and Methods

### 2.1. Reagents

All cell culture reagents were purchased from Sigma Chemical Co. (St. Louis, MO, USA). Antibodies against MAPKs, NF*κ*B, iNOS, and actin were purchased form Cell Signaling Technology (Beverley, MA, USA). Reagents for qPCR were purchased from Promega (Madison, WI, USA) and Roche Applied Science (USA).

### 2.2. Cell Lines

The BV2 cell line was obtained from Dr. Luc Vallieres, Quebec City, Canada. Immortalization of the BV2 cell line via infection of murine primary microglial cell cultures with a v-raf/v-myc oncogene-carrying retrovirus (J2) has been described previously [22]. Phenotypically, BV2 cells tested positive for MAC1 and MAC2 antigens. BV2 cells were maintained in Dulbecco's modified essential medium supplemented with 10% heat-inactivated fetal bovine serum, streptomycin (10 mg/mL), and penicillin (10 U/mL) at 37°C. The dopaminergic neuronal cell line used in the *in vitro* neuronal viability studies, N27, is derived from embryonic rat dopaminergic mesencephalic neurons via SV40 large T-antigen immortalization. The cells were grown in RPMI medium 1640 containing 10% fetal bovine serum, penicillin (100 units/mL), and streptomycin (100 *μ*g/mL) [23]. To examine the effects of DHB, microglial BV2 cells were treated with DHB for 1 h before stimulation with LPS. Cell viability was determined by 3-(4,5-dimethylthiazol-2-yl)-2,5-diphenyltetrazolium bromide reduction assay as previously described [24].

### 2.3. Mice

10-week-old C57BL/6 male mice (Jackson Laboratories, Bar Harbor, ME) were used for the described *in vivo* studies. Mice were housed according to standard animal care protocols, fed ad libitum, kept on a 12 h light/dark cycle, and maintained in a pathogen-free environment in the Buck Institute Vivarium. All experiments were approved by local IACUC review and conducted according to current NIH policies on the use of animals in research. For DHB studies (*n* = 10 per group), the drug was diluted to a final dose of 100 mg/kg (in 5% ethanol) and administered intraperitoneally to mice 6 h prior to 2 consecutive intraperitoneal injections of either saline vehicle or 20 mg/kg of MPTP given 12 h apart [21]. Age-matched controls (10 weeks of age) were also treated with 2 × 20 mg/kg MPTP or saline, 12 h apart. Seven days following the final MPTP or saline injection, mice were sacrificed for either tissue harvest for biochemical assays or brain fixation via intracardiac perfusion for immunohistochemistry.

### 2.4. RT-PCR

BV2 cells (7.5 × 10^5^ cells on a 6 cm dish) were treated with LPS in the presence or absence of DHB and total RNA extracted with TRI reagent. Total RNA (1 *μ*g) was then reverse-transcribed in a reaction mixture containing 1 U RNase inhibitor, 500 ng random primers, 3 mM MgCl_2_, 0.5 mM dNTP, and 10 U reverse transcriptase in RT buffer (Promega). The synthesized cDNA was used as a template for qPCR analysis using the universal probe library system from Roche. For *in vivo* studies, striatal tissue was dissected out from various treatment groups, total RNA extracted using the TRIZOL method, and reverse-transcribed to cDNA. qPCR analysis of GAPDH, TNF-*α*, IL-6, iNOS, and HO-1 was performed using the Roche universal probe library detection system. Relative quantification of gene expression was performed using the comparative threshold (CT) method. Changes in mRNA expression level were calculated following normalization to GAPDH. The ratios obtained after normalization are expressed as fold change over corresponding wild-type controls [25].

### 2.5. Measurement of IL-6 and Nitrate Levels in Conditioned Media (CM)

Microglial cells (1 × 10^5^ cells per well in a 24-well plate) were pretreated with DHB or normal media for 1 h and then stimulated with LPS (100 ng/mL). CM was collected from the cultured microglia 24 h following LPS stimulation and the concentrations of IL-6 measured using the mouse IL-6 ELISA kit from BD Biosciences according to manufacturer's instructions [26]. Accumulated nitrite was measured in the CM using the Griess reagent (Sigma). For mouse IL-6 measurements, striatal tissue was dissected from saline or MPTP groups in the absence or presence of co-DHB treatment and IL-6 levels measured from tissue homogenates using the ELISA kit as described above. For HO-1 inhibition studies, BV2 were pretreated with ZnPPIX (10 *μ*M) for 30 min and treated with DHB for another 1 h before LPS application. The NO levels were measured after 24 h following LPS stimulation.

### 2.6. Western Blot Analyses

Whole cell protein lysates from BV2 cells were prepared in lysis buffer, protein samples separated by 10% sodium dodecyl sulfate-polyacrylamide gel electrophoresis, and transferred to nitrocellulose membranes (Invitrogen). Membranes were blocked with 5% skim milk in 10 mM Tris-HCl containing 150 mM NaCl and 0.5% Tween 20 (TBST), then incubated with primary antibodies (1 : 1000) against iNOS, phosphorylated 65 subunit, phosphorylated p38, phosphorylated JNK, p38, JNK, HO-1, or actin [27]. After thoroughly washing with TBST, horseradish peroxidase-conjugated secondary antibodies (1 : 3000 dilution in TBST; Millipore, CA, USA) were applied and blots developed using an enhanced chemiluminescence detection kit (Pierce Biotechnology, Rockford, IL, USA).

### 2.7. Measurement of Intracellular Reactive Oxygen Species (ROS) Levels

Intracellular accumulation of ROS was measured using H2DCF-DA (Sigma) as previously described [28]. In brief, microglial cells were stimulated with LPS for 16 h in the absence or presence of DHB then stained with 20 *μ*M H2DCF-DA in Hank's balanced salt solution buffer for 1 h at 37°C. DCF fluorescence intensity was measured on a fluorescence plate reader at 485 nm excitation and 535 nm emission (Molecular Devices, CA).

### 2.8. Transient Transfection of NF*κ*B Reporter Construct and Assay by Luciferase

Transfection of the NF*κ*B binding reporter gene into BV2 cells was performed using lipofectamine 2000 (Invitrogen, USA). The NF*κ*B binding reporter plasmid contains three copies of the *κ*B-binding sequence fused to the firefly luciferase gene (Clontech, Mountain View, CA, USA). BV2 cells (2 × 10^5^ cells per well in a 12-well plate) were transfected with 1 *μ*g of the reporter construct mixed with lipofectamine 2000. After 48 h, cells were harvested and luciferase activity assayed as previously described [28]. To determine the effect of DHB on reporter gene activity, cells were pretreated for 1 h with the agent before treating with LPS (0.1 *μ*g/mL) for 4 h prior to cell harvest.

### 2.9. Immunocytofluorescent Staining to Assess Nuclear Translocation of NF*κ*B

BV2 cells were seeded onto glass coverslips and stimulated with LPS (100 ng/mL) following pretreatment with DHB (100 *μ*M) or media for 1 h. Then cells were fixed in 4% paraformaldehyde, permeabilized in 0.5% Trition X-100 for 30 min. After blocking with 5% nonfat milk in PBS buffer, cells were incubated with rabbit anti-p65 antibodies for 1 h at room temperature. After a brief wash, cells were incubated with Alexa fluor-conjugated secondary antibody (1: 500, Molecular Probes) [28]. Finally, the cells were washed again, mounted with vectashield hard mount with DAPI and visualized using a Zeiss LSM 510 confocal microscope.

### 2.10. Assay of Effects of CM from BV2 on Cell Viability in Dopaminergic N27 Cells

BV2 cells were stimulated with LPS (100 ng/mL) in the absence or presence or DHB (100 *μ*M) for 24 h. CM from control, DHB treated, LPS-treated, or LPS + DHB-treated cells was added to dopaminergic N27 cells plated in 96 well plates. After 48 h, N27 cell viability was assessed via the MTT assay.

### 2.11. *In Vivo* Immunohistochemistry

Immunochemistry was performed on sections from the striata of fixed perfused brains from mice treated with MPTP in the absence or presence of DHB cotreatment versus saline-treated controls. Microglial activation was detected using primary antibodies against Iba1 [29]. Briefly, 7 *μ*m SN sections from paraffin-embedded brains were cut and processed for staining. Sections were mounted onto slides and processed in 10 mM citrate buffer for enhancement of antigen retrieval. After blocking with 10% donkey serum for 1 h, primary antibody (1 : 500, anti-Iba1 antibody; Dako) was applied to the sections for overnight incubation at 4°C followed by biotinylated secondary antibody and 3,3′-diaminobenzidine processing.

### 2.12. Statistical Analysis

 Unless otherwise stated, all experiments were performed in triplicate samples and repeated at least three times. The data are presented as the mean ± SE and statistical comparisons between groups were performed using one-way ANOVA followed by Student's *t*-test between two populations based on the assumption that both populations have normal distribution. A *P* value < 0.05 was considered significant.

## 3. Results

### 3.1. DHB Suppresses LPS-Mediated Upregulation of Both Proinflammatory Genes and Associated Gene Products in BV2 Microglial Cells

**Figure 1:**
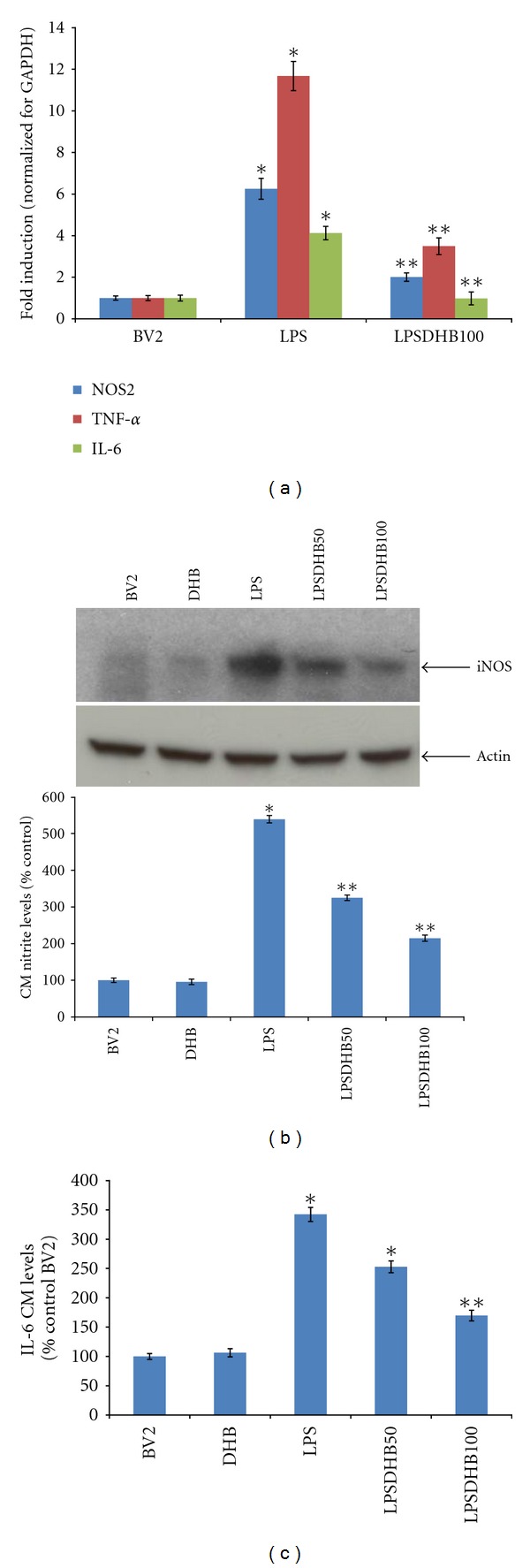
(a) BV2 microglial cells were pretreated with DHB (100 *μ*M) for 1 h followed by cotreatment with LPS (100 ng/mL) for 6 h. Total RNA was isolated and real-time PCR analysis performed. Relative mRNA levels for iNOS, TNF-*α*, and IL-6 were normalized for GAPDH. Each bar represents means ± SEM. *P < 0.05 compared with the control group, **P < 0.05 compared with the LPS-treated group. (b) Protein was extracted from whole cell lysates and subjected to western blot analysis for iNOS protein levels; actin was used as a loading control. Conditioned media (CM) was collected after 24 h LPS (100 ng/mL) ± DHB treatment and nitrite levels determined using the Griess reagent. Data are presented as means ± SD of at least four independent experiments. *P < 0.05 compared with the control group, **P < 0.05 compared with the LPS treated group. (c) IL-6 levels in the CM were determined using an ELISA kit according to manufacturer's recommendations. Data are presented as means ± SD of at least four independent experiments. *P < 0.05 compared with the control group, **P < 0.01 compared with the LPS treated group.

LPS stimulation is known to induce the expression of multiple proinflammatory genes that can in turn contribute to neuroinflammation and subsequent neurotoxicity. In our studies, LPS treatment of BV2 microglial cells was found to result in upregulation of mRNA levels for the proinflammatory cytokines TNF-*α* and IL-6 and for the nitric oxide (NO) producing enzyme iNOS (Figure 1(a)). Pretreatment with DHB not only significantly decreased proinflammatory gene expression but also iNOS protein levels and amounts of NO and IL-6 secreted into the CM in a concentration-dependent manner (Figures 1(b) and 1(c)). DHB at the concentrations used for these studies (10 to 100 *μ*M) were not found to have any effects on cell viability as assessed by the MTT assay (data not shown).

### 3.2. DHB Attenuates LPS-Induced Intracellular ROS Production and Activation of NF*κ* B and MAP Kinase Pathways

**Figure 2:**
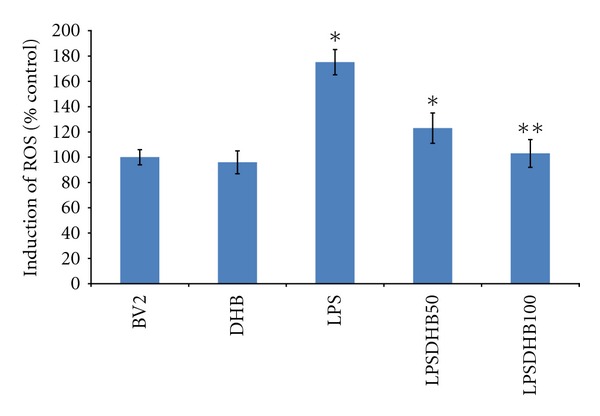
BV2 microglial cells were pretreated with DHB for 1 h followed by co-treatment with LPS (100 ng/mL) for 16 h. ROS levels were measured using the DCFDA method. *P < 0.05 compared with the control group, **P < 0.05 compared with the LPS-treated group.

**Figure 3:**
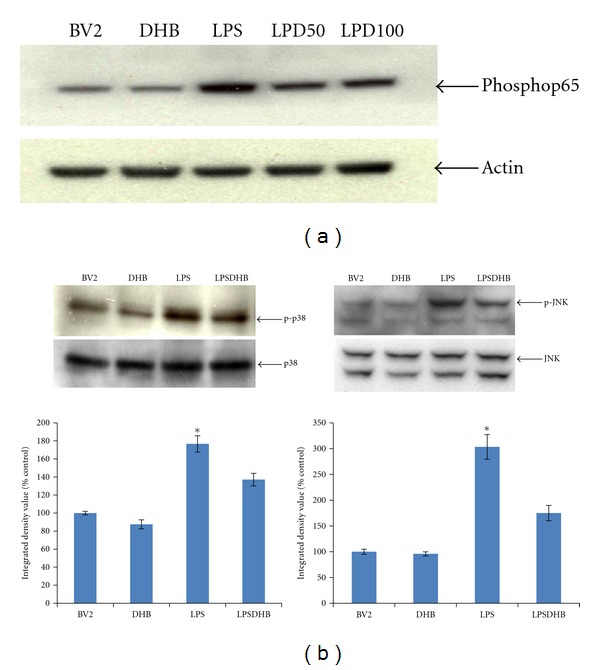
BV2 cells were pretreated with DHB for 1 h (50 & 100 *μ*M) and then stimulated with LPS (100 ng/mL) for a 1 h incubation period. Cells were lysed, run on a SDS-PAGE gel, transferred to PVDF membranes, and blotted with specific antibodies to (a) phosphorylated p65 NF*κ*B subunit or (b) phosphorylated or unphosphorylated p38 and JNK. Actin was used as a loading control. Band density (integrated density value) is expressed graphically as a percentage ratio of densitometric optical density of phosphorylated forms to that of nonphosphorylated p38 and JNK. Data (mean ± SD) are from three independent experiments; *P < 0.05 relative to untreated control sample.

**Figure 4:**
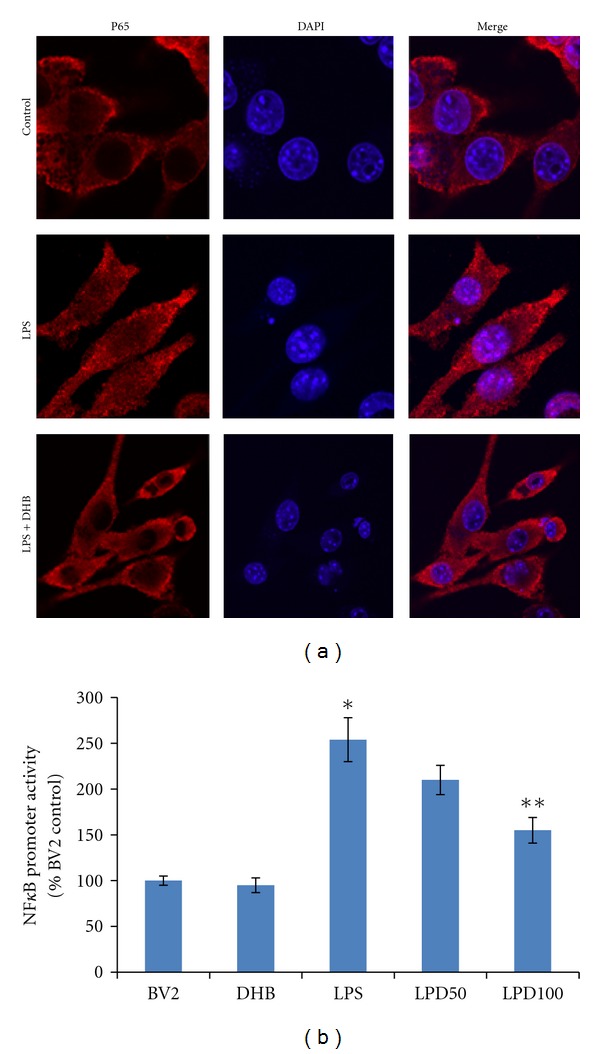
(a) After pretreatment with DHB (100 *μ*M) for 1 h, BV2 cells were stimulated with 100 ng/mL LPS for 1 h and nuclear translocation of the NF*κ*B p65 subunit assessed by confocal fluorescence microscopy using a fluorescent anti-p65 antibody. Representative laser confocal microcopy images of p65 (red stain) and nuclear DAPI staining (blue) in cells exposed to LPS ± DHB are shown; pink, merged. (b) BV2 cells were transiently transfected with NF*κ*B-Luc for 24 h and treated with 100 ng/mL LPS for 4 h ± DHB for 1 h. Cell lysates were assayed for luciferase activity (mean ± SE, *n* = 4). *P < 0.05 versus control, **P < 0.05 versus LPS.

LPS is known to induce the production of intracellular ROS that can in turn stimulate expression of proinflammatory genes via the activation of secondary messenger systems including NF*κ*B and the MAP kinases [30]. LPS exposure was found to not only induce increased intracellular ROS production in microglial BV2 cells (Figure 2), but also phosphorylation of the p65 subunit of NF*κ*B and the MAP kinases JNK and p38 necessary for their activation (Figure 3). Pretreatment with DHB was found to significantly block LPS-induced ROS production in a dose-dependent manner (Figure 2) and to modulate LPS-induced phosphorylation of these pathway components (Figure 3). Furthermore, it prevented nuclear translocation of p65 and subsequent NF*κ*B-dependent transcriptional activity, the latter in a dosage-dependent fashion (Figure 4). These data suggest that DHB can prevent oxidative stress-mediated induction of these inflammatory pathways.

### 3.3. DHB Induces Upregulation of Hemoxygenase-1 (HO-1) Expression and Protein Levels in BV-2 Microglia Cells

**Figure 5:**
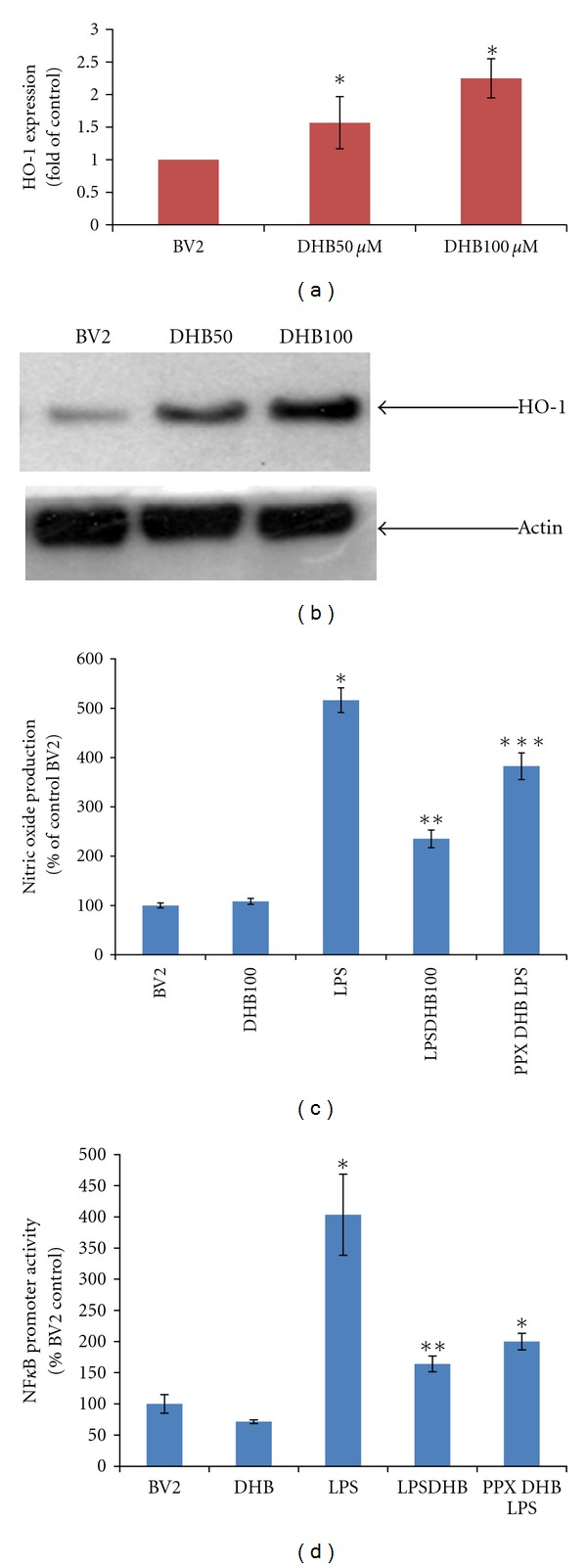
(a) Cells were stimulated with given concentrations of DHB for 6 h and mRNA levels of HO-1 analyzed via real-time PCR as described. (b) BV2 cells were stimulated with various concentrations of DHB for 24 h and whole cell lysates subjected to western blot analysis using antibody against HO-1 protein; *P < 0.05 versus control. (c) Cells were pretreated with ZnPPIX (20 *μ*M) for 30 min followed by co-treatment with DHB (100 *μ*M) for another 1 h before LPS (100 ng/mL) addition. Conditioned media (CM) was collected after 24 h of LPS treatment and nitrite levels were determined using the Griess reagent. Each bar represents mean ± SEM from at least four independent experiments. *P < 0.05 compared with the control group, **P < 0.05 compared with the LPS treated group, ***P < 0.05 compared with the LPSDHB100 treated group. (d) BV2 NF*κ*B-Luc cells were pretreated with HO-1 inhibitor ZnPPIX (20 *μ*M) for 30 min followed by co-treatment with DHB (100 *μ*M) for another 1 h before LPS (100 ng/mL) addition for 4 h. Cell lysates were assayed for luciferase activity (mean ± SE, *n* = 4). *P < 0.05 versus control, **P < 0.05 versus LPS.

We demonstrated in our previous study that PHD inhibition via DHB results in upregulation of the enzyme HO-1, likely via a HIF-1*α* transcription-dependent process [21]. HO-1 has recently emerged as a key molecule in the resolution of oxidative stress-mediated microglial activation including that induced by LPS [31]. BV2 microglia treated with DHB for 24 h were found to have both elevated HO-1 expression and protein levels and this was found to occur in a concentration-dependent manner (Figures 5(a) and 5(b)). Pretreatment with ZnPPIX, a potent HO-1 inhibitor, was found to abrogate the inhibitory effects of DHB on LPS-induced NO production (Figure 5(c)) have a small but significant effects on NFkB activity (Figure 5(d)).

### 3.4. The Neuroprotective Effects of DHB Coincides with Inhibition of Microglial Activation Both *In Vitro* and *In Vivo*

**Figure 6:**
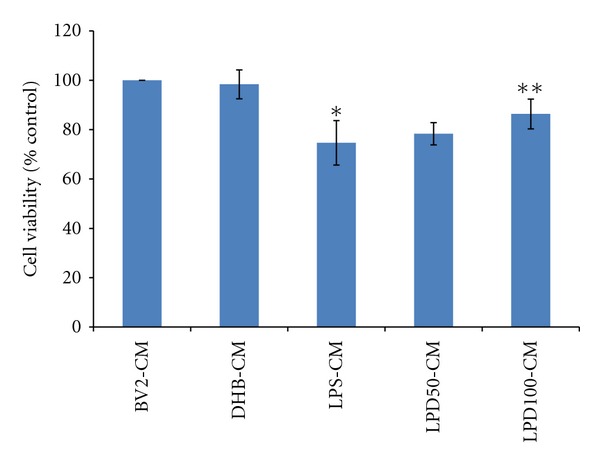
BV2 cells were stimulated with LPS (100 ng/mL) ± DHB (100 *μ*M) for 24 h. CM from control (BV2-CM), DHB treated (DHB-CM), LPS-treated (LPS-CM), and LPS/DHB treated (50 & 100 *μ*M; LPD50-CM, LDP100-CM) BV2 cells was added to dopaminergic N27 cells plated in 96 well plates. After 48 h, N27 cell viability was assessed via the MTT assay. Data is expressed as mean ± SD, *n* = 4. *P < 0.01, compared with control-CM group, **P < 0.01, compared with LPS-CM group.

A number of studies have demonstrated that activated microglia can induce neural toxicity [32–34]. Our *in vitro* results suggest that DHB could possibly exert neuroprotective effects via its ability to suppress microglial activation. To assess this possibility, the neuronal toxicity of conditioned media (CM) from LPS-treated BV2 cells grown in the absence and presence of DHB treatment was evaluated in dopaminergic N27 cells. CM from LPS-stimulated microglia (LPS-CM) was found to produce significant toxicity in N27 cells, which was attenuated in CM from BV2 cells grown in the additional presence of co-DHB treatment (Figure 6). This suggested that the toxicity of the CM derived from LPS-treated microglia could be dependent on release of soluble neurotoxic factors and be prevented by presence of DHB. We cannot, however, rule out the possibility that part of the neuroprotective effects may be due to residual DHB in the CM from the microglial cells that could have a direct impact on neuronal survival.

**Figure 7:**
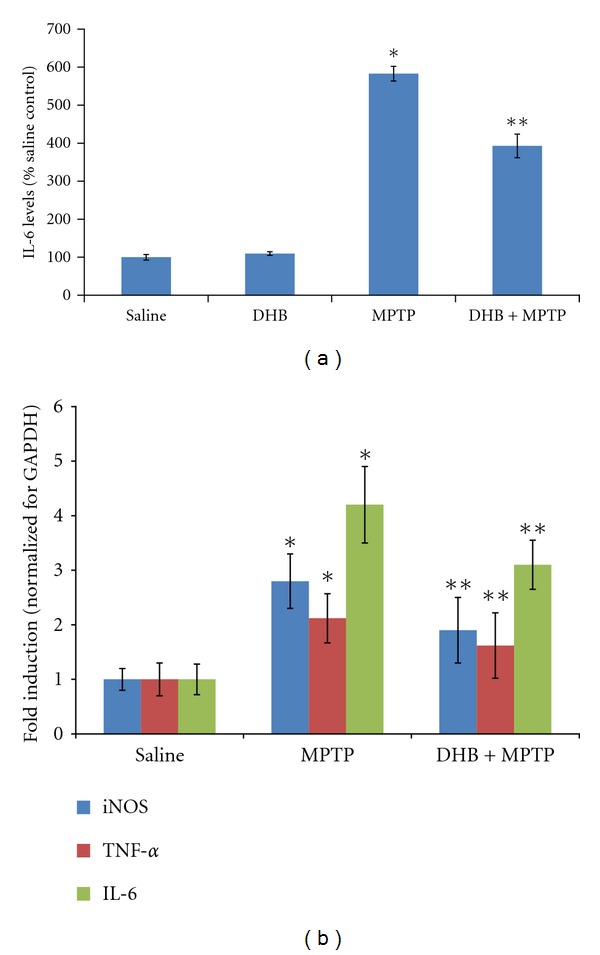
Mice were pretreated with DHB (100 mg/kg, intraperitoneally) 6 h prior to administration of either saline (Sal) or MPTP (20 mg/kg x2, 12 h apart). Animals were sacrificed 2 days later. (a) Levels of striatal IL-6 were measured using an ELISA kit from BD Biosciences. Each bar represents mean + SEM for 4 animals in each group. *P < 0.05 compared with the Sal group, **P < 0.01 compared with the MPTP-treated group. (b) DHB pretreatment decreases the mRNA levels of MPTP upregulated proinflammatory cytokines. *P < 0.05 compared with the Sal group, **P < 0.05 compared with the MPTP-treated group.

**Figure 8:**
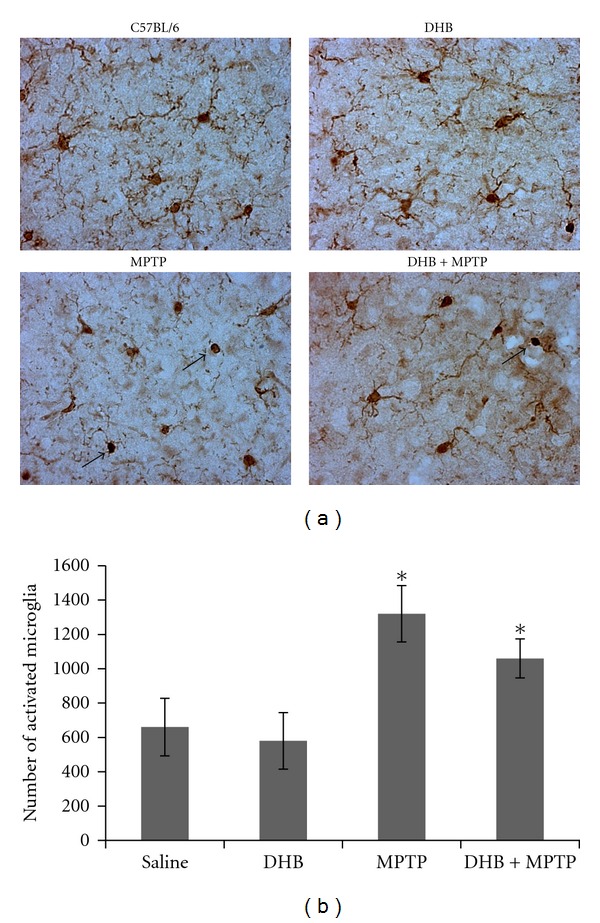
Iba1 immunostaining of SN sections of mice treated with either saline (Control), DHB, MPTP or DHB + MPTP. (a) Microglia in MPTP-treated mice display a morphological transformation from resting ramified microglia to activated amoeboid like forms with withdrawn processes (indicated by arrows) which is inhibited in the presence of DHB treatment. (b) MPTP treatment leads to a significant increase in the number of activated microglia relative to saline and DHB treated mice. Activated microglia were bilaterally counted under a 40x objective. Data are means ± SEM for 4 mice per group, *P < 0.05 compared to saline group.

Based on our *in vitro *results, we next assessed the ability of DHB to prevent microglial activation in MPTP-treated mice, a commonly used toxin model of PD. Our previously published data demonstrated that DHB treatment prevented nigrostriatal neurotoxicity associated with this model [21]. We found that MPTP treatment resulted in a significant increase in striatal IL-6 levels that was significantly attenuated by co-DHB administration (Figure 7(a)). We next evaluated the effects of DHB treatment on striatal expression of various cytokine mRNAs (TNF*α*, IL6, and iNOS) following MPTP intoxication. qPCR analysis revealed significant induction of proinflammatory gene expression in the presence of MPTP that was blocked by pretreatment with DHB (Figure 7(b)). To assess the impact of DHB on microglial activation in MPTP-treated mice [28], immunohistochemistry was performed on sections from control and MPTP mice in the absence and presence of DHB cotreatment using the microglial activation marker Iba1 (Figure 8). Prior to activation, microglia normally exhibits a highly ramified morphology (as observed in both control and DHB-treated mice). In response to activation by MPTP, microglia begins to withdraw their ramified branches and became amoeboid-like. This activated morphological phenotype was found to be significantly attenuated in the presence of DHB pretreatment, demonstrating the ability of DHB to in part attenuate microglial activation resulting from MPTP treatment.

## 4. Discussion

Previous studies have demonstrated that the pharmacological PHD inhibitor 3,4-DHB can directly protect cultured neuronal cells against both oxidative stress [20] and ischemic injury [19]. Our current study suggests that in addition to these direct effects, DHB could also elicit neuroprotection via a heretofore unknown action of the drug—its capacity to dampen microglial activation via its ability to prevent oxidative induction of the MAPK and NF*κ*B signaling pathways. These pathways can in turn elicit the synthesis and release of proinflammatory factors from activated microglia, indirectly impacting on neighboring neurons [35–37].

LPS is known to induce oxidative phosphorylation of p38 and JNK within microglia, stimulating activation of NF*κ*B via phosphorylation of its p65 subunit. Subsequent nuclear translocation of p65 results in increased expression of several proinflammatory genes [28]. In our current study, DHB treatment was found to partially inhibit LPS-induced increases in microglial ROS levels as well as phosphorylation of p38 and JNK, subsequent p65 translocation and mediation of transcriptional activation. This was associated with reduced expression of proinflammatory genes in microglial cells induced either directly *in vitro* by LPS or *in vivo* by MPTP along with reduced release of IL-6. *In vitro*, DHB also partially prevented both increased NO release and had a small but significant effect on NF*κ*B activation. Pharmacological inhibition of iNOS has been shown to prevent dopaminergic neurodegeneration as a consequence of microglial activation and transgenic mice lacking iNOS are more resistant to MPTP-mediated dopaminergic neurotoxicity [38–41].

The anti-inflammatory effects of DHB could be due to its ability to upregulate HO-1 expression. HO-1 has recently been demonstrated to inhibit oxidatively induced microglial activation such as that elicited by LPS [31]. In the present study, inhibition of HO-1 using the pharmacological inhibitor ZnPPIX was found to attenuate DHB-mediated inhibition of microglial NO production as well as having a small but significant effect on NF*κ*B activity. HO-1 is a microsomal enzyme that catalyzes oxidative cleavage of the porphyrin ring of the heme molecule leading to the formation of biliverdin, carbon monoxide (CO), and free iron [42]. The beneficial protective effects of HO-1 in inflammation are mediated not only via enzymatic degradation of proinflammatory free heme, but also via production of the antiinflammatory compounds bilirubin and carbon monoxide [43, 44]. Recent reports have suggested that upregulation of HO-1 may have both anti-oxidant and anti-inflammatory effects [30, 45–47] and that therefore the HO-1 system may be an important therapeutic target for inflammation associated with neurodegeneration. The concomitant enhancement of HO-1 expression and reduction in LPS-induced NO production by DHB is consistent with previous reports using other anti-inflammatory agents [48, 49].

Based on our current results, we propose that the neuroprotective effects of DHB may be in part due to its ability to inhibit the inflammatory NF*κ*B/cytokine pathway. However, this does not definitively rule out that it may alternatively be acting via direct neuroprotective effects on the neurons themselves. Future studies using *in vivo* LPS-induced nigrostriatal degeneration models will be helpful in delineating the relative contributions of anti-inflammatory versus direct antioxidant in terms of the neuroprotective effects of DHB.

Microglial activation has long been associated with dopaminergic neuropathology in PD [9, 32]. DHB administration was found to reduce microglial activation and the release of soluble inflammatory factors in association with reduced neurotoxicity both *in vitro* and *in vivo*. DHB's ability to dampen microglial activation may suggest a novel mechanism of action for the drug that mechanistically could involve HO-1 induction. This hypothesis will require further validation, but presents a possible novel mode of action for the drug.
